# Effectiveness of Unfocused vs. Focal Shock Waves Combined with Botulinum Toxin on Spasticity in Brain-Damaged Patients

**DOI:** 10.3390/toxins17050209

**Published:** 2025-04-22

**Authors:** Antonio Déniz, Pedro Saavedra, Isabel Marrero, Samuel Barrera, Raúl Domínguez, Raúl Mendoza, Jorge Rodríguez

**Affiliations:** 1 Neurorehabilitation, Rehabilitation Service of the University Hospital of Gran Canaria Dr. Negrín, 35010 Las Palmas, Spain; antonio.deniz@ulpgc.es (A.D.); samubr@outlook.com (S.B.); rauldguezguerra@gmail.com (R.D.); rmenmenv@gobiernodecanarias.org (R.M.); jrodriguez40206@gmail.com (J.R.); 2 Department of Mathematics, University of Las Palmas de Gran Canaria, 35017 Las Palmas, Spain; pedro.saavedra@ulpgc.es; 3 Physiology, Department of Biochemistry and Molecular Biology, University of Las Palmas de Gran Canaria, 35016 Las Palmas, Spain

**Keywords:** extracorporeal shock wave therapy, focal shock waves, unfocused shock waves, botulinum toxin type A, spasticity, stroke, multiple sclerosis

## Abstract

Spasticity is a common complication in patients with neurological disorders, increasing disability and hampering quality of life. Combined treatment with focused shock waves (fESWT) and botulinum toxin (BoNT-A) has been shown to increase the effectiveness and duration of the effect of BoNT-A on spasticity in patients with brain injuries. We studied the effectiveness of the combination of unfocused shock waves (uESWT) and BoNT-A on spasticity. This prospective study with systematic randomization included 24 patients with brain injury, a small sample size due to clinical limitations, and spasticity was measured using the Ashworth scale (AS) for those with lower limb involvement; gait speed was assessed using the 10-m gait test (10MWT). To judge patient satisfaction with treatment, we used the Consumer Reports Effectiveness Scale-4 (CRES-4). Both uESWT and fESWT with BoNT-A resulted in a 3-point improvement in the median spasticity score, which was maintained from week 2 to week 26, and a statistically significant reduction in the 10MWT was observed. Furthermore, the proportion of patients who were very satisfied with the treatment was higher with uESWT (91%) than with fESWT (69.2%). To the authors’ knowledge, this is the first study to evaluate the added benefit of concurrent and combined treatment with uESWT and BoNT-A injections to improve spasticity in patients with stroke or multiple sclerosis and show higher patient satisfaction with the treatment.

## 1. Introduction

Spasticity is a frequent complication in neurological diseases with brain damage, which increases disability and reduces the quality of life of affected patients. It poses a great clinical challenge. Its therapeutic approach requires a complex and multidisciplinary treatment due to its effects on the organism [1].

In brain-damaged patients, spasticity mainly affects the muscles of the upper and lower limbs and spine. When it affects the upper limb, the flexor muscles are predisposed, on the other hand, if it affects the lower limb, the extensor muscles tend to be affected [2]. Hence, causing great disability [1]. In addition, spasticity can cause pain, joint limitation, contractures, and pressure ulcers [3]. This leads to a significant impairment of the individual’s functionality, significantly impacting the patient’s ability to perform daily activities. This results in a reduction in their quality of life and an increase in dependency. It generates higher expenses, up to four times the costs compared to patients without spasticity [4], a substantial economic burden for the health care system.

The neurological pathologies that most commonly generate spasticity are stroke and multiple sclerosis (MS). It is estimated to affect 20–40% of stroke survivors [5] within 12 months of stroke onset and 60–90% of people with multiple sclerosis [6].

Various medical procedures are available to treat spasticity, such as botulinum toxin injections applied locally to specific muscle groups, oral antispasmodic drugs, physiotherapy, nerve blocks, etc. [7,8,9]. However, the management of spasticity remains a challenge due to the adverse effects of these treatments. Botulinum toxin injections are the gold standard in the management of spasticity [7]. Work by blocking the release of acetylcholine at the nerve endings, but it must be repeated every 4 months and can stimulate the formation of neutralizing antibodies, leading to a reduction in the effectiveness of treatments [7,10,11]. Antispasmodic drugs, such as baclofen and tizanidine, can reduce muscle strength and cause sedation and drowsiness, which worsen the functional status of these patients and their cooperation in rehabilitation treatment. Additionally, doses of these drugs often need to be increased, as pharmacological tolerance may develop after prolonged use [12]. Baclofen also carries the risk of overdose and withdrawal [13]. Therefore, there is a need to find a therapy that improves the effectiveness and duration of the effect of botulinum toxin in reducing spasticity and provides greater safety in the management of these patients.

Over the last years, extensive research has shown that extracorporeal shock wave therapy (ESWT) on spasticity [14,15,16,17,18] is a safe, effective, and non-invasive treatment for stroke and MS patients [18,19,20,21,22].

ESWT in patients with spasticity may reduce muscle tone in spastic muscles, although their mechanism of action is currently unknown. We know that it reduces the stiffness of muscle tissue as measured by elastosonography in patients with cerebral palsy; they may change muscle elasticity [23]. Studies in rats have shown that ESWT destroys endplates at the neuromuscular junction, reduces acetylcholine receptors, and decreases the maximum compound muscle action potential amplitude, reducing muscle contraction [24].

Therefore, ESWT should be considered an initial therapeutic option in patients with spasticity.

Depending on the energy source, we distinguish between electrohydraulic, electromagnetic, and piezoelectric focal shock waves [4,10,25]. Focal ESWT (fESWT) concentrates the energy at a specific point in the tissue and can reach higher energy levels than unfocused ESWT (uESWT).

uESWT can be piezoelectric and electrohydraulic, generating pulses that are not concentrated at a particular point but are dispersed evenly over a larger area. The uESWT probes would facilitate the treatment of spastic muscle, allowing for a more homogeneous treatment [26,27]. However, they can trigger the same biological responses as targeted devices, eliminating energy concentrations at one point [28]. uESWT is better tolerated by the patient [29].

Previous studies in stroke patients [29] have demonstrated the benefit of combining the treatment of botulinum toxin and focal shock waves in reducing spasticity. Furthermore, in stroke and MS patients [30], a longer duration of muscle tone reduction was noted.

The effect of uESWT associated with BoNT-A injection on spasticity in stroke and multiple sclerosis patients is currently unknown. We hypothesize that they would also improve and prolong the BoNT-A effect on spasticity, likely fESWT, as well as increase patient satisfaction.

## 2. Results

**Figure 1:**
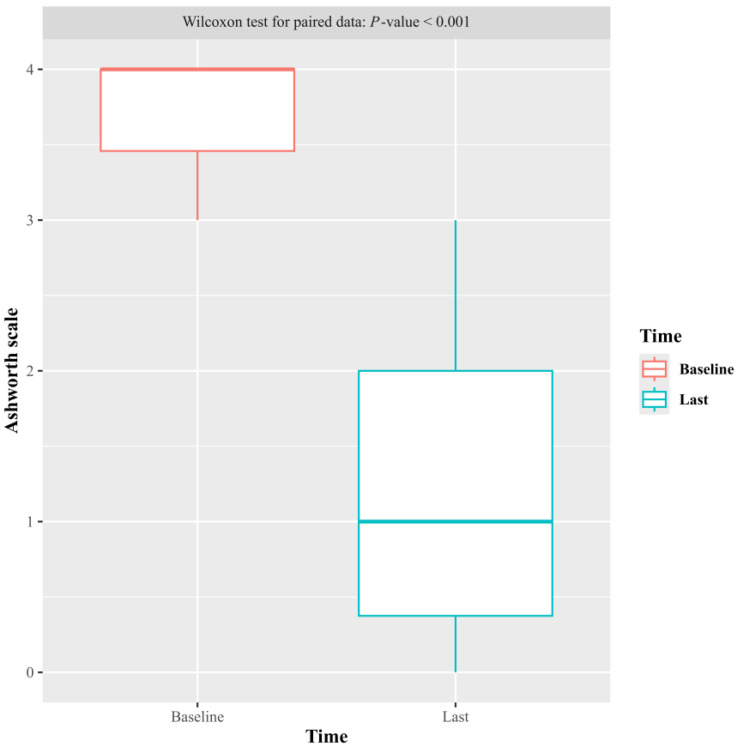
Distribution of the Ashworth scale at baseline and the last follow-up determination for each patient.

There was a statistically significant reduction in spasticity, which correlated with a lower score on the Ashworth scale (AS) of the affected upper or lower limb musculature (Figure 1). The reduction in spasticity occurred from the first week and was maintained until the 26th week of follow-up (Table 1). The reduction in spasticity from baseline to the last follow-up determination for each patient was 3 points on the AS (from 4 to 1) (Figure 1), with *p* < 0.001.

**Figure 2:**
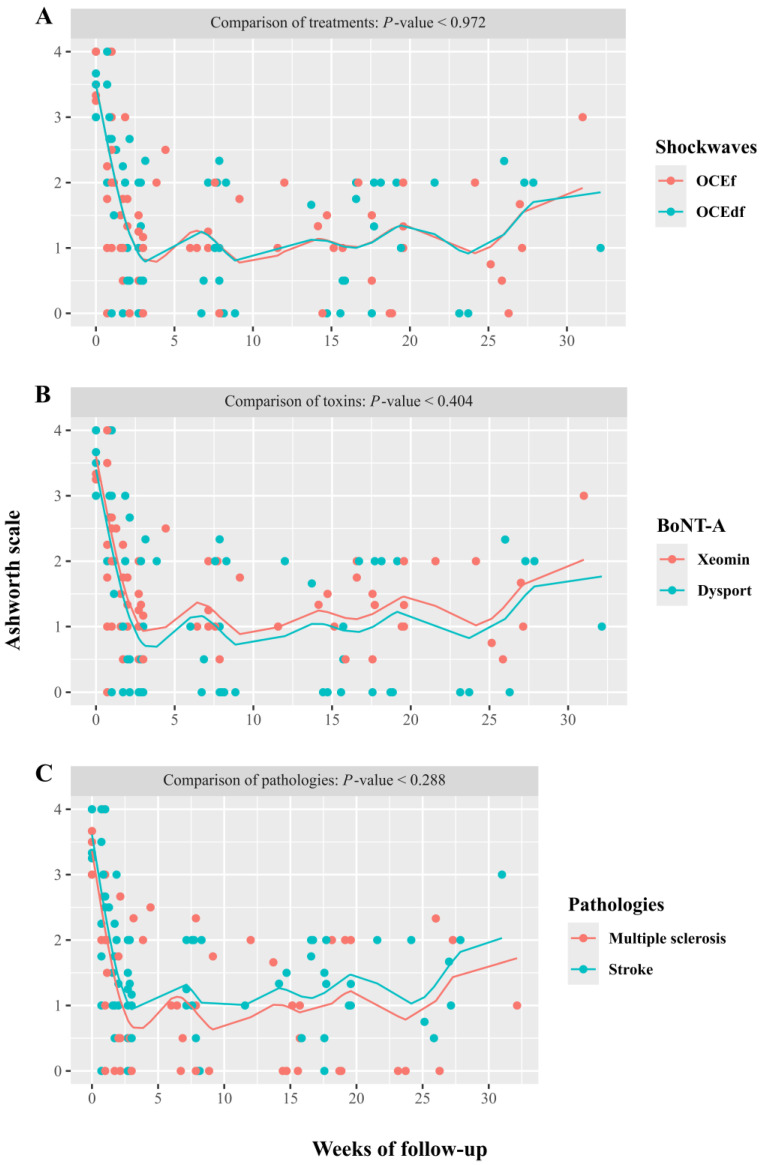
Progress of the spasticity assessed by the Ashworth scale score according to each factor: (**A**). Shock wave. (**B**). Toxin. (**C**). Pathologies. In all cases, the effects on spasticity were statistically significant (*p* < 0.001).

This improvement in spasticity was statistically significant and independent of the type of ESWT used (fESWT or uESWT), the toxin injected (Xeomin^®^ or Dysport^®^), or the pathology (multiple sclerosis or stroke) (Figure 2). There were no statistically significant differences in the reduction in spasticity and duration of the effect between shock wave treatments, the toxins used, or the cause of spasticity treated (Figure 2). However, due to the sample size, this is only exploratory.

**Figure 3:**
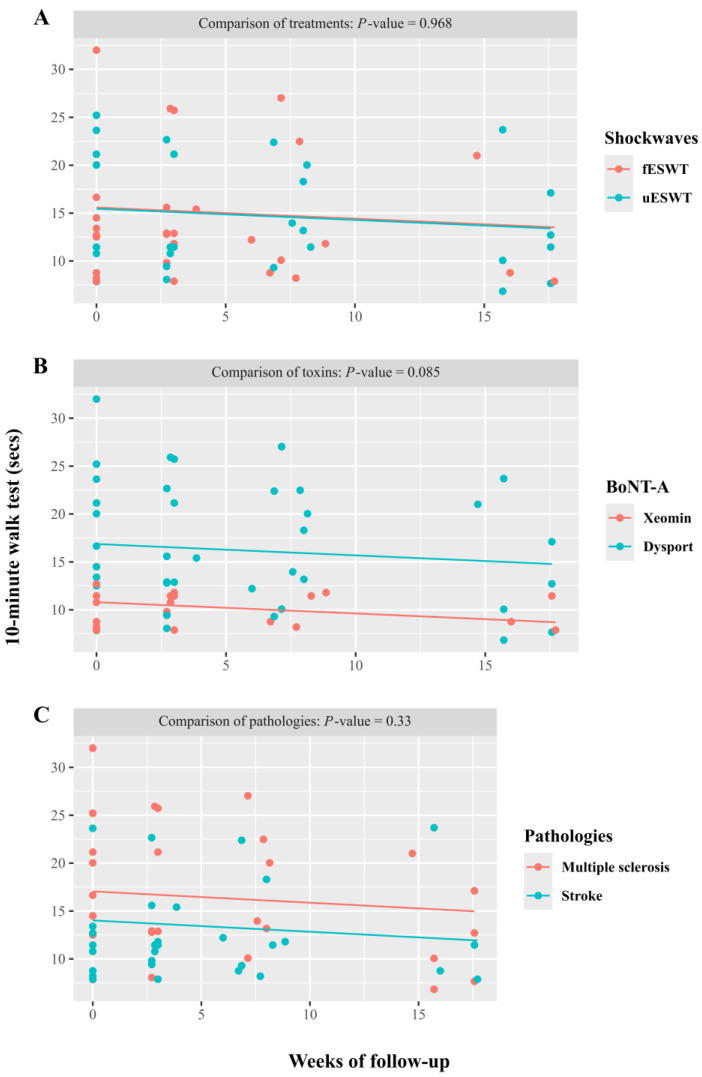
Progress of the 10-m walk test (10MWT) according to each factor: (**A**). Shock waves. (**B**). Botulinum toxin (BoNT-A). (**C**). Pathologies. In all cases, the effects of 10MWT improvement were statistically significant (*p* < 0.001).

On the other hand, those patients with lower limb spasticity were assessed by the 10-m walk test (10MWT). A reduction in execution time was observed in both those treated with fESWT or uESWT, as well as with the toxins Xeomin^®^ and Dysport^®^ for both pathologies (MS or stroke) (Figure 3). In all cases, the results were statistically significant. However, due to the sample size, this is underpowered.

The speed improvement in the 10MWT was independent of the ESWT used, the toxin used, or the spasticity etiology. There were no statistically significant differences between shock wave treatments, the toxins used, or the causes of brain damage.

These effects can be seen in the clinical evolution of the patients and shown in the videos (Supplementary Materials Videos S1–S4) of patient 15, with MS and spastic paresis of the left lower limb, who used an orthosis for assisted hip flexion due to psoas weakness. Treated in the left triceps surae, the gait pattern and gait speed improved from the third week, with improvement progressing from weeks 17 to 24.

**Figure 4:**
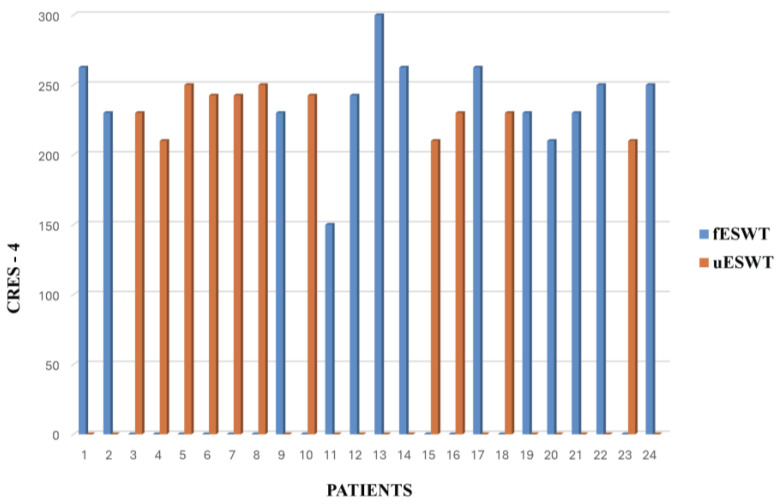
Treatment efficacy: patient-perceived efficacy of combined treatment assessed with CRES-4. fESWT, or uESWT associated with BoNT-A therapy on spasticity in stroke or MS patients.

Regarding the treatment efficacy perceived by the patients, we assessed it with the Consumer Reports Effectiveness Scale-4 (CRES-4) after applying the third session of fESWT and uESWT. All patients presented a score between 200 and 300 on this scale, indicating a very good perception of the improvement of spasticity with both uESWT and fESWT except for patient 11 treated with fESWT, who perceived it as good (Figure 4).

**Figure 5:**
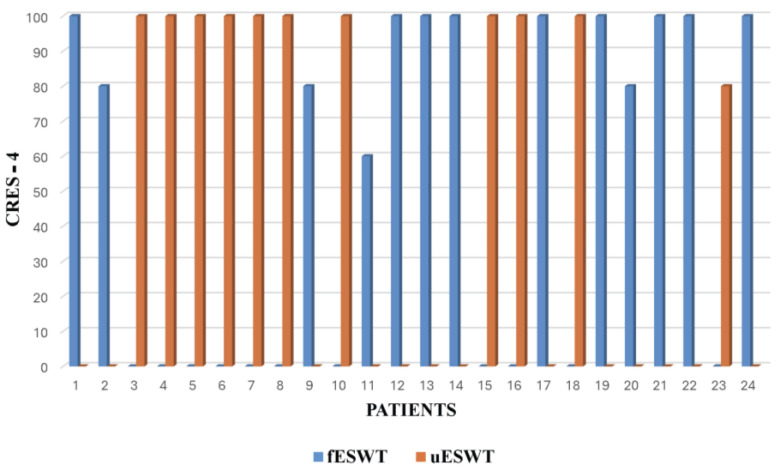
Evaluation of patient satisfaction with the combined treatment received was assessed with the first item of the CRES-4. ESWT used fESWT or uESWT.

In terms of patient satisfaction with the treatment received, we assessed it with the first item of the CRES-4. 69.2% of the patients treated with fESWT were very satisfied, compared with 91% of the patients treated with uESWT (Figure 5).

## 3. Discussion

To the author’s knowledge, this is the first study simultaneously combining BoNT-A and uESWT for the treatment of spasticity in patients with brain damage secondary to stroke or MS, with a follow-up period of 6 months which we observed a 3-point improvement in spasticity in Ashworth scale from the second week of treatment which was maintained until the 26th week of follow-up. To date, there are few studies that combine the treatment with BoNT-A and ESWT in spasticity [23,29,31,32,33], of which only four have been carried out in patients with brain damage due to stroke or MS [29,30,31,32].

uESWT has been used to treat pathologies such as plantar fasciitis [27]; however, in spasticity, we only have evidence with fESWT and radial extracorporeal shock wave therapy (rESWT).

The SBOTE study [29] was the first study to combine BoNT-A and fESWT in stroke patients and compare its effectiveness with BoNT-A and electrostimulation. The results showed that fESWT were superior to electrostimulation in enhancing the effect of BoNT-A on spasticity reduction, improving it by 2 points in AS, one point less than our work; they only performed a 3-month follow-up. In a 2021 study conducted in MS patients [32], BoNT-A was associated with rESWT, not simultaneously, 4 months after BoNT-A treatment, with a follow-up of 3 months after the end of rESWT. They observed a reduction in spasticity of less than 1 point in modified AS and prolonged the reduction up to one month after the last session.

A more recent study [31] of randomized controlled trials in stroke patients showed a two-point reduction on the modified Ashworth scale in the finger flexor muscles that was maintained for up to three months in patients treated with fESWT associated with BoNT-A, but not in those treated with BoNT-A alone. Another 2024 study [30] compared the application of BoNT-A alone versus the combined treatment of BoNT-A and fESWT on patients with spasticity secondary to stroke or MS. In this investigation, the additional effect of fESWT on spasticity was observed, with a reduction in the Ashworth scale of two points, and the effect was prolonged until the sixth month of follow-up. In addition, an improvement in function in the lower limb of the treated patients was observed as evidenced by a progressive reduction in the 10-m walk test, which increased the patients’ walking speed.

Our study extends the current evidence by demonstrating that BoNT-A-associated uESWT has similar results in reducing spasticity to fESWT combined with BoNT-A.

Regarding the mechanism of action of BoNT-A, it has been shown to reduce spasticity by presynaptically inhibiting the release of acetylcholine at the neuromuscular junction [34,35]. However, the mechanism of ESWT on spasticity remains unclear. In a study in Sprague Dawley rats [36] applying a session of rESWT to the gastrocnemius muscle of the left paw, with low energy like the one used in our study, they observed by scanning electron microscopy destruction of the motor endplates at the neuromuscular junction without affecting muscle fibres or axon terminals [36], reducing acetylcholine receptors and compound muscle action potential amplitude, without affecting peripheral nerve conduction [24].

This is in addition to the changes that ESWT produce in muscle rheology and elasticity, as suggested in previous studies [23], where a reduction in muscle stiffness was observed by sonoelastography in cerebral palsy patients treated with BoNT-A and low-energy fESWT but not in those treated with BoNT-A alone, suggesting that fESWT may change muscle elasticity. The previous studies [29,30] and our results, where we observed that uESWT demonstrate comparable efficacy to fESWT when combined with BoNT-A, increasing the efficacy of the toxin in reducing spasticity and prolonging its effect up to 6 months, could support the findings of Kenmoku et al. [24,36] on the mechanism of action of shock waves. Thus, the postsynaptic effect on the neuromuscular junction, reducing acetylcholine receptors, would be added to the already known presynaptic effect of BoNT-A.

Furthermore, the latter could be improved if we take into account intramuscular neural arborization [37,38,39] when we infiltrate the toxin into the spastic muscle, as these would be the areas with the highest density of motor endplates.

Also, the satisfaction of patients with the treatment received is an issue that takes importance, and CRES-4 is a tool to judge it. Patients similarly perceived the efficacy of treatment with both types of ESWT. However, the percentage of patients who were very satisfied was higher in patients treated with uESWT. This may be due to the fact that, unlike fESWT, uESWT facilitates a more homogeneous application of energy in the muscle, reaching a greater number of endplates and allowing us to select the depth of application, preventing the maximum energy from reaching the periosteum of the bones, making them more tolerable for our patients.

### Limitations

The study has the following limitations. The small sample size. Spasticity was only assessed using the Ashworth scale, and the evaluator was the same person who applied the treatment. We did not evaluate functional changes in the upper limb, nor the intensity of the rehabilitation treatment. Finally, we did not take intramuscular neural arborization into account in the infiltrated muscles. This could improve the presynaptic effects of the combined treatment on spasticity and is a target for future research.

## 4. Conclusions

This is the first study to demonstrate that uESWT associated with BoNT-A has an effect similar to the association of fESWT with BoNT-A that increases the effectiveness of the latter in reducing spasticity and improving gait speed in patients treated in the lower extremity, prolonging these effects over time. Furthermore, patient satisfaction with uESWT was higher than with fESWT, the former being the most suitable for use in the combined treatment of spasticity. These results would support a summation of the effects of the toxin at the presynaptic level and of the focal and unfocused shock wave at the postsynaptic level in the neuromuscular junction. However, further research with a greater number of patients and with a better understanding of intramuscular neural arborization could confirm these results and improve the effectiveness of combined treatment at the presynaptic level.

## 5. Materials and Methods

This is a prospective, systematic randomization study carried out between December 2023 and May 2024 in outpatients who gave their informed consent and had all their doubts clarified in accordance with the Declaration of Helsinki, all prior to the start of the study. It was approved by the Medicines Research Ethics Committee (CEIm).

The study had a follow-up period of 6 months, including 24 adult patients aged between 20 and 70 years with brain damage secondary to a stroke (11 patients) or multiple sclerosis (13 patients) (Table 2), with a chronic evolution and spasticity on the Ashworth scale of 2, 3, or 4 in the affected muscles. The patients were able to collaborate with the rehabilitation treatment.

**Figure 6:**
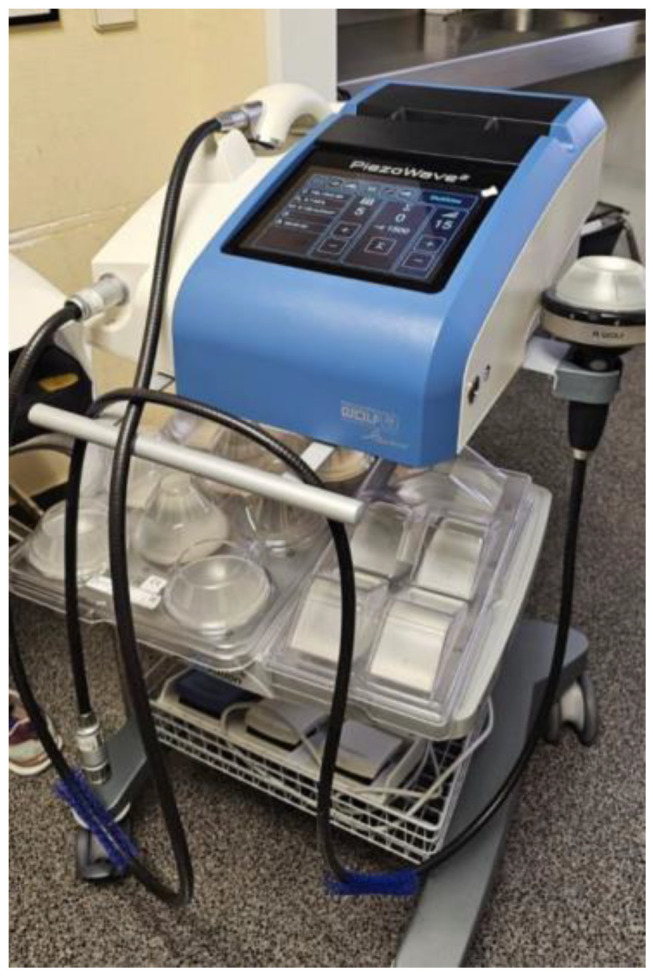
Piezowave2T Touch WOLF appliance.

The ESWT treatment was carried out with a Piezowave2T Touch WOLF device (Figure 6): applying fESWT (FB10 G6 probe) and uESWT [FBL10 × 5 G2 probe with gel pad adapters (5, 10, 15 and 20 mm) so that the pulses reach the desired depth and allowed us to treat the muscle in a more homogeneous way]. An energy flux density (EFD) of 0.1 mJ/mm^2^ and 1500 pulses at a frequency of 5 Hz was used, which was based on previous studies of treatments with isolated [19,21,22] or combined [23,24,29,30] focal shock waves on spasticity with BoNT-A, taking into account that the biological effects of shock waves are similar with the same energy densities.

**Figure 7:**
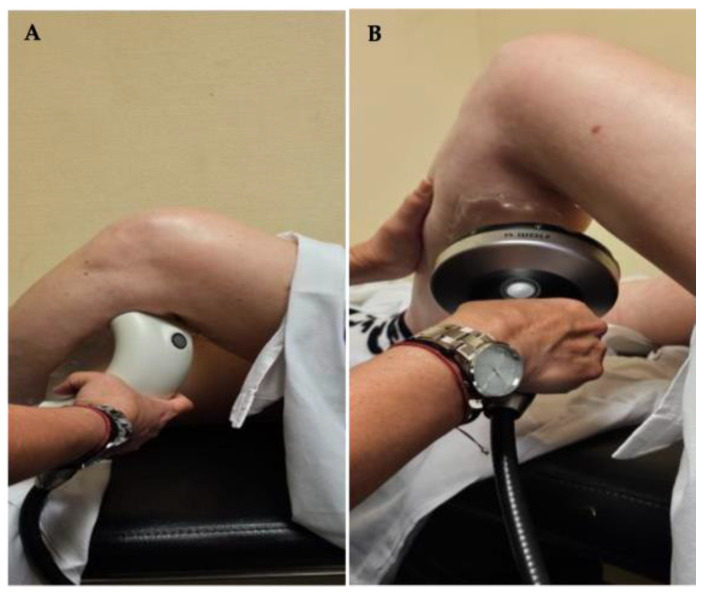
(**A**). Application of uESWT to triceps surae. (**B**). Application of fESWT on triceps surae.

They were applied on the pectoralis major, subscapularis, upper limb (elbow flexors, pronator teres, flexor pollicis longus, wrist or finger flexors) and lower limb (rectus femoris, adductors, hamstrings, triceps surae or tibialis posterior) (Figure 7), depending on the spasticity of each patient.

This fESWT and uESWT regimen was added to the patient’s usual BoNT-A treatment, using incobotulinumtoxin A (Xeomin^®^ from MERZ, Dessau, Germany) in the upper limb and abobotulinumtoxin A (Dysport^®^ from IPSEN, Paris, France) in the lower limb, applied at the optimal doses [40].

We use Xeomin^®^ and Dysport^®^ in this way according to institutional protocol.

For greater safety and precision, it was guided by a Samsung H7/XH7 ultrasound scanner (Samsung Medison CO., Gangwon-do, Republic of Korea) with linear probes of Fr = 14 Hz and 18 Hz, which also allowed us to determine the depth of each muscle and to use the appropriate gel pad on the uESWT probe to focus the pulses on the treated muscle.

At the first visit (week 0), we applied BoNT-A, and at week one, the first session of fESWT versus uESWT, including 13 patients for fESWT and 11 for uESWT (Table 2). Weekly (Table 3), we continued with two sessions of fESWT or uESWT. In addition, all patients received rehabilitative treatment during the treatment and the follow-up period.

Patients were observed at baseline and, after the intervention, were followed up to a maximum of 26 weeks. Therefore, repeated measures are available for each patient. Follow-up times were obtained exactly as the time elapsed from baseline to each of the controls and were expressed in weeks.

For the statistical analysis, the following variables were assessed: spasticity was assessed by the Ashworth scale (AS), and in those with lower limb spasticity, the 10-m walk test (10MWT) was performed. The AS measures muscle resistance during passive stretching with values ranging from 0 to 4, with 0 indicating no increase in muscle tone and 4 indicating the impossibility of mobilizing the affected joint [41]. The same doctor administered the treatments and evaluated the spasticity.

The 10MWT assesses how long it takes the patient to walk 10 m in a straight line, measured in seconds [42].

The analysis was carried out before and after each session to evaluate spasticity. The 10MWT was conducted before and after the third session. Subsequently, follow-up was performed one month after the start and at three and six months thereafter (Table 2).

### Statistical Analysis

Categorical variables are expressed as frequencies and percentages and as median and interquartile range (IQR = 25th–75th percentile). As this is a repeated measures study, the data were fitted using mixed models. Thus, to assess the evolution of each marker (Ashworth scale and 10-m walk test) as a function of each factor (shock wave, toxin and etiology), an additive mixed model was considered, which has the form [43]: *Marker = μ + s (Week) + φ_factor + Patient + e.* Here, *Week* denotes the week of follow-up (*Week* = 0 corresponds to baseline), *s (Week)* is a non-linear function of the week, which is estimated using cubic splines, *φ_Factor* is the factor effect, *Patient* is the random effect of the patient, and *e* is the within-patient variability.

Statistical significance was set at *p* < 0.05. Data were analyzed using the R package, version 4.2.1 (R Development Core Team, 2022) [44].

Patient acceptance and satisfaction with the fESWT versus uESWT were assessed after the third application of ESWT, by means of the application of the scale of satisfaction with the treatment received (CRES-4) [45,46] as a novel tool in populations with spasticity.

**Figure 8:**
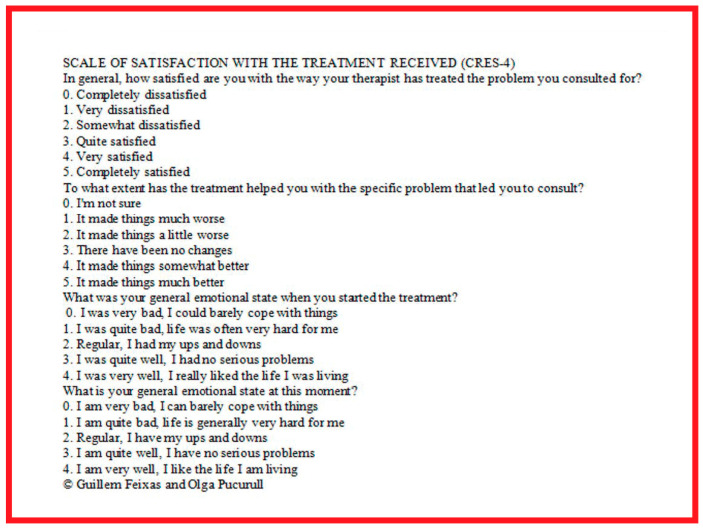
Consumer Reports Effectiveness Scale-4 (CRES-4).

It assesses the effectiveness of the treatment as perceived by the patient (Figure 8) by means of 4 items that assess, respectively: satisfaction, solution of the problem and perception of emotional change with the treatment. The score is calculated: *CRES-4 = (20 × Satisfaction) + (20 × Problem Solving) + [12.5 × (4 + current emotional state–pre-treatment emotional state)] = 0–300.*

The higher the total score, the greater the effectiveness of the treatment according to the patient [47]^.^ Patient satisfaction is assessed with the first item of the scale (Figure 8) and is scored from 0 to 5; after calculation, the score would be 0–100; the higher the score, the higher the degree of satisfaction.

## Figures and Tables

**Table 1:** Variations in spasticity on the Ashworth Scale with the combined treatments.

BoNT-A + fESWT			
Basal Preinjection	5 Weeks After Inyection	12 Weeks After Injection	26 Semanas After Injection
μ + σ 3.57 ± 0.57	1.17 ± 0.64	1.07 ± 0.69	0.86 ± 0.74
		**BoNT-A + uESWT**	
μ + σ 3.66 ± 0.42	1 ± 0.77	1.01 ± 0.85	1.28 ± 0.88

**Table 2:** Characteristics of the patients.

Characteristic		Value *
Age (years)		57.8 (52.3; 67.7)
Male		12 (50.0)
Female		12 (50.0)
*Shock waves*	fESWT uESWT	13 (54.2)
		11 (45.8)
*Toxin*	Xeomin^®^ Dysport^®^	11 (45.8)
		13 (54.2)
*Pathologies*	Multiple sclerosis	11 (45.8)
	Stroke	13 (54.2)

* Data are medians (IQR) and frequencies (%).

**Table 3:** Combined treatment regimen and observation period.

Week	Treatment	Observation
0	BoNT-A	Predose
1	1st session fESWT/uESWT	
2	2nd session fESWT/uESWT	Effect 1st session
3	3rd session fESWT/uESWT	Effect 2nd session
5	-	Effect 3rd session
13	-	Effect 3rd session
25	-	Effect 3rd session

## Data Availability

The original contributions presented in this study are included in the article/Supplementary Material. Further inquiries can be directed to the corresponding author(s).

## Supplementary Materials

The following supporting information can be downloaded at https://www.mdpi.com/article/10.3390/toxins17050209/s1; Video S1: Patient 15, Pretreatment; Video S2: Patient 15, three weeks post BoNTA + uESWT; Video S3: Patient 15, seventeen weeks post BoNTA + uESWT; Video S4: Patient 15, twenty-four weeks post BoNTA + uESWT.
